# Supplementary material for: The Influence of Physical Fields (Magnetic and Electric) and LASER Exposure on the Composition and Bioactivity of Cinnamon Bark, Patchouli, and Geranium Essential Oils
Source: Plants (Basel). 2024 Jul 21;13(14):1992. doi: 10.3390/plants13141992 (PMC11281253; doi:10.3390/plants13141992)
Supplement: Supplementary file 1 [file plants-13-01992-s001.zip › Chromatograms of the Essential Oils.pdf]

Chromatograms of the Tested Essential Oils

1. Chromatograms of the Cinnamon Essential Oils (*Cinnamomum zeylanicum* Blume)

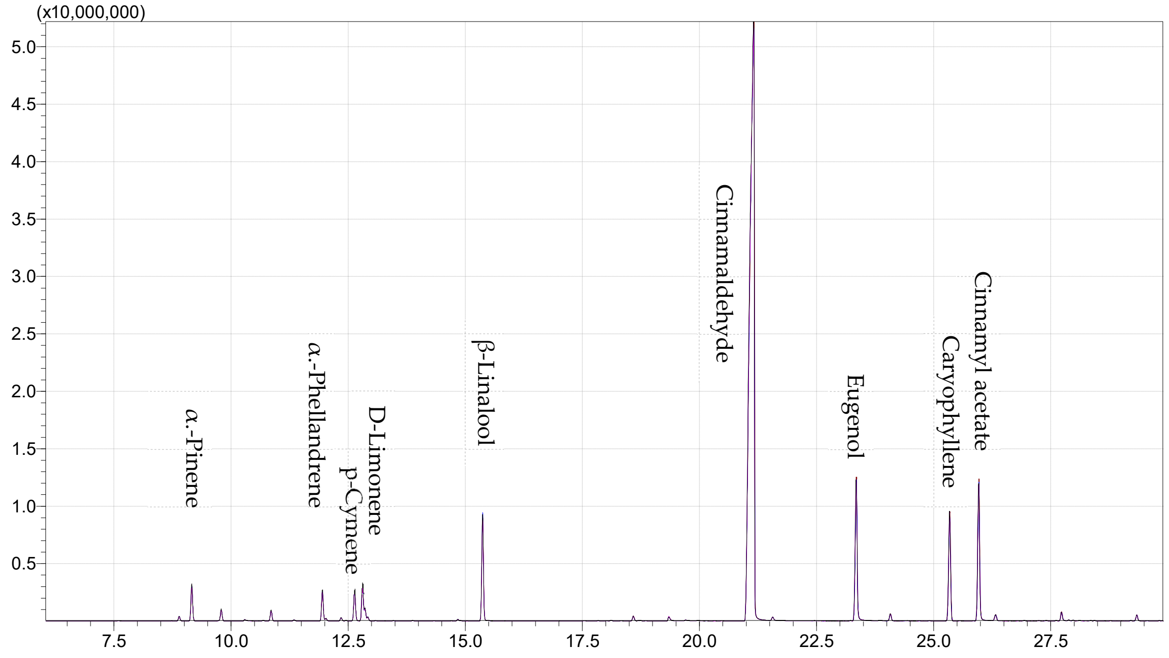

Legend:

- Black – CEO
- Pink – CEOL
- Blue – CEOM
- Brown - CEOEl

2. Chromatograms of the Patchouli Essential Oils (*Pogostemon cablin* (Blanco) Benth.)

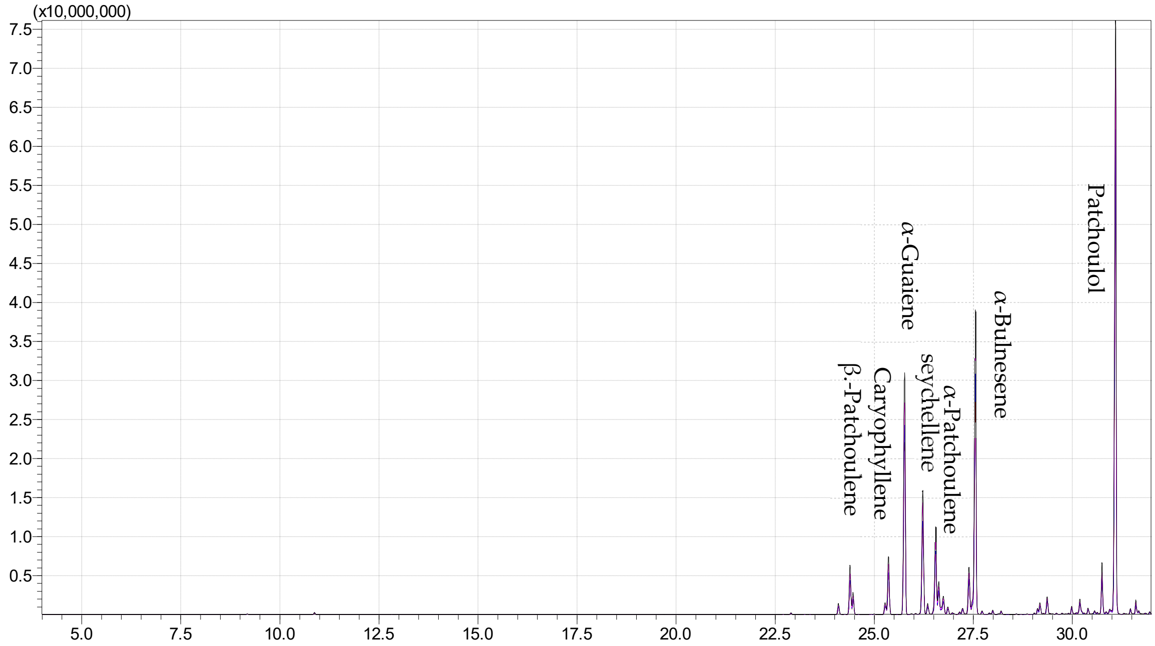

Legend:

- Black – PEO
- Pink – PEOL
- Blue – PEOM
- Brown - PEOEl

3. Chromatograms of the Geranium Essential Oils (*Pelargonium graveolens* L'Hér.)

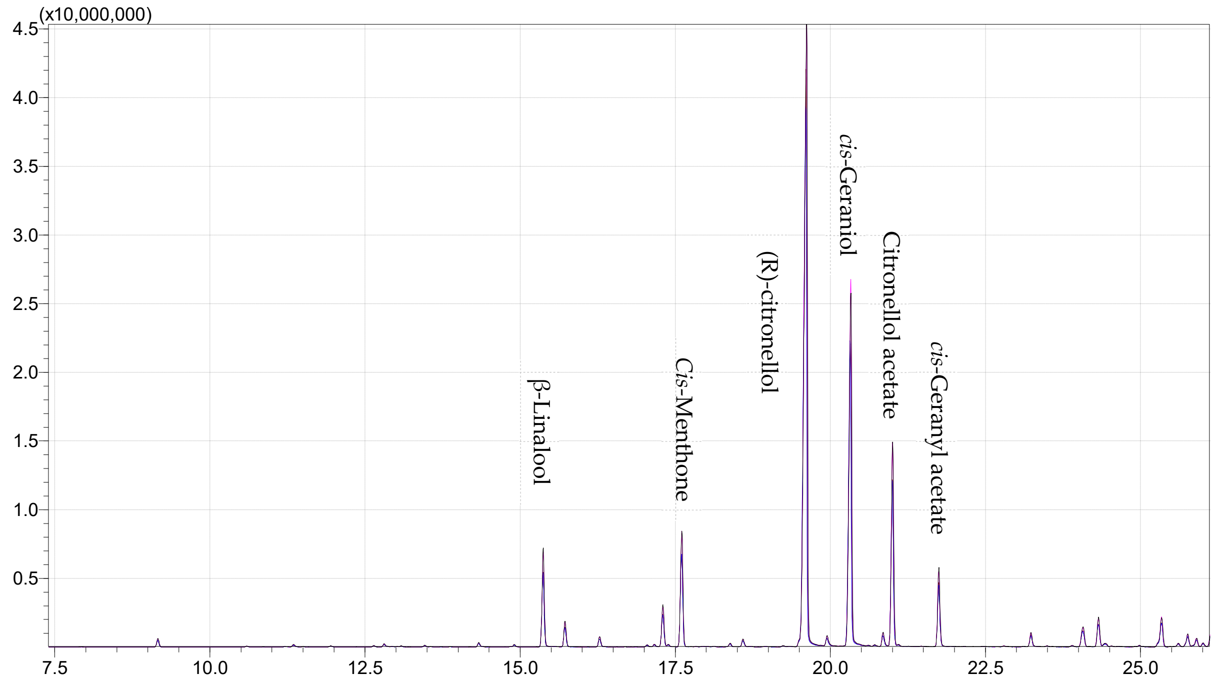

Legend:

- Black – GEO
- Pink – GEOL
- Blue – GEOM
- Brown - GEOEl
