# Supplementary material for: The Influence of Physical Fields (Magnetic and Electric) and LASER Exposure on the Composition and Bioactivity of Cinnamon Bark, Patchouli, and Geranium Essential Oils
Source: Plants (Basel). 2024 Jul 21;13(14):1992. doi: 10.3390/plants13141992 (PMC11281253; doi:10.3390/plants13141992)
Supplement: Supplementary file 1 [file plants-13-01992-s001.zip › Table S1í¬List of abbreviations.pdf]

Table S1 - List of abbreviations

The following table describes the significance of abbreviations used throughout the manuscript, along with the page on which each one is defined or used for the first time. The most commonly used abbreviations for unit measure were not listed, but they are defined in the manuscript.

No. crt	Abbreviation	Definition	Page
1	**EO**	essential oil	1
2	**MEL**	physical fields (magnetic and electric) and LASER irradiation	1
3	SMF	static magnetic field	2
4	CEO	cinnamon bark essential oil	3
5	CEOM	cinnamon bark essential oil exposed to magnetic field	3
6	CEOL	cinnamon bark essential oil exposed to laser irradiation	3
7	CEOEl	cinnamon bark essential oil exposed to electric field	3
8	PEO	patchouli essential oil	5
9	PEOM	patchouli essential oil exposed to magnetic field	5
10	PEOEl	patchouli essential oil exposed to electric field	5
11	PEOL	patchouli essential oil exposed to laser irradiation	5
12	GEO	geranium essential oil	6
13	GEOL	geranium essential oil exposed to laser irradiation	6
14	GEOM	geranium essential oil exposed to magnetic field	7
15	GEOEL	geranium essential oil exposed to electric field	7
16	MIC	minimum inhibitory concentration	8
17	MBC	minimum bactericidal concentration	8
18	DPPH	2,2-Diphenyl-1-picrylhydrazyl	10
19	$ABTS^+$	2,2′-Azinobis-(3-Ethylbenzthiazolin-6-Sulfonic Acid	10
